# High glucose enhances lipopolysaccharide‐induced inflammation in cultured BV2 microglial cell line

**DOI:** 10.1002/iid3.610

**Published:** 2022-04-19

**Authors:** Hao‐Chang Hung, Sheng‐Feng Tsai, Shih‐Ren Sie, Yu‐Min Kuo

**Affiliations:** ^1^ Division of Endocrinology and Metabolism, Department of Internal Medicine Kaohsiung Veterans General Hospital Kaohsiung Taiwan; ^2^ Institute of Basic Medical Sciences, College of Medicine National Cheng Kung University Tainan Taiwan; ^3^ Department of Cell Biology and Anatomy, College of Medicine National Cheng Kung University Tainan Taiwan; ^4^ Department of Anesthesiology Ditmanson Medical Foundation Chia‐Yi Christian Hospital Chiayi Taiwan

**Keywords:** diabetes, neuroinflammation, NF‐κB, p38, TLR4

## Abstract

**Introduction:**

Diabetes mellitus emerges as a global health crisis and is related to the development of neurodegenerative diseases. Microglia, a population of macrophages‐like cells, govern immune defense in the central nervous system. Activated microglia are known to play active roles in the pathogenesis of neurodegenerative diseases.

**Methods:**

This study aimed to investigate the effects of high glucose on low‐dose lipopolysaccharide (LPS)‐induced activations of inflammation‐related signaling molecules in cultured BV2 microglial cells.

**Results:**

Compared to cells cultured in the normal glucose medium (NGM, 5.5 mM), the LPS‐induced activation of NF‐κB lasted longer in cells cultured in high glucose medium (HGM, 25 mM). HGM also enhanced the expression of inducible nitric oxide synthase (iNOS). Among the mitogen‐activated protein kinases, HGM enhanced the LPS‐induced phosphorylation of p38 without affecting the phosphorylation of Erk1/2 or JNK. BV2 cells cultured in HGM expressed higher levels of TLR4 than those cells cultured in NGM.

**Conclusion:**

High glucose aggravated LPS‐induced inflammatory responses of microglia via enhancing the TLR4/p38 pathway and prolonging the activation of NF‐κB/iNOS signaling. Controlling blood glucose levels is advised to manage neuroinflammation and related neurodegenerative diseases.

## Graphical abstract

High glucose prolongs LPS‐induced phosphorylation of NF‐kB.

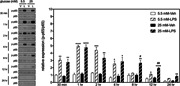

## 1 INTRODUCTION

Hyperglycemia is the most prominent sign of diabetes mellitus and is considered a critical factor for the development of chronic inflammation. In healthy subjects, ingestion of 75 g of glucose elevates the blood glucose levels and induces oxidative stress by increasing the expression of p47phox, a component of NADPH oxidase, and the generation of leukocyte‐derived reactive oxygen species.^1^ Increased free radicals subsequently trigger the inflammatory signaling mediated by redox‐sensitive transcription factors, such as NF‐κB.^2^, ^3^, ^4^ The pro‐inflammatory feature of glucose with similar mechanisms is also evident in monocytes and endothelial cells.^5^, ^6^, ^7^

Epidemiological analyses reveal high comorbidity between diabetes mellitus and neuroinflammation‐related neurodegenerative diseases,^8^, ^9^, ^10^ suggesting that high blood glucose may contribute to neuroinflammation. Neuroinflammation is highly mediated by microglia, a population of glial cells that are resident macrophage‐like immune cells in the central nervous system. In physiological conditions, microglia are under a resting state characterized by a ramified morphology.^11^, ^12^ Upon facing stimulatory signals, microglia undergo varied extents of activation, such as morphological changes and producing inflammatory mediators.^12^ It has been shown that glucose level in the culture medium affects the activity and inflammatory status of microglia.^13^, ^14^ Interestingly, high glucose aggravates the lipopolysaccharide (LPS)‐induced releases of pro‐inflammatory cytokines in microglia in a TLR4‐dependent manner.^14^ TLR4 is a pattern‐recognition receptor recognizing distinct pathogen‐associated molecular patterns, such as LPS.^15^ Upon ligand binding, TRL4 initiates downstream NF‐κB and mitogen‐activated protein (MAP) kinase signaling pathways.^16^, ^17^ However, whether and how high glucose level affects the LPS‐induced signaling cascades in microglia remain unclear.

Herein, we cultured BV2 microglial cells in normal glucose medium (NGM) and high glucose medium (HGM) and determined the temporal profiles of LPS‐induced activation of NF‐κB and MAP kinase signaling pathways in these cultures by examining the phosphorylation degrees of p65 (one of the components that form NF‐κB), p38, Erk1/2, and JNK. The expressions of iNOS, a major downstream pro‐inflammatory meditator regulated by NF‐κB, were also examined.

## 2 MATERIALS AND METHODS

### 2.1 BV2 microglial cultures and treatment

Immortalized murine microglial BV2 cells (RRID: CVCL_0182) were cultured in either HGM (high‐glucose [25 mM or 450 mg/dl] Dulbecco's modified Eagle's medium [DMEM], Cat#: 21969035, Thermo Fisher Scientific) or NGM (low‐glucose [5.5 mM or 99 mg/dl] DMEM, Cat#: 10567022, Thermo Fisher Scientific). The media were supplemented with 10% fetal bovine serum, containing extremely low endotoxin (<0.05 EU/ml; Lot#: VP2002200, Cat#: TMS‐013‐BKR, Merck‐Millipore), GlutaMAX™ (Cat#: 35050079, Thermo Fisher Scientific), and penicillin‐streptomycin (Cat#: 15140122, Thermo Fisher Scientific). The cultures were maintained in a humidified atmosphere of 5% CO_2_ and 95% air at 37°C, and subcultures were performed when cell density reached 80% confluence (about every 2–3 days).

The BV2 cells were seeded in six‐well culture plates with a density of 3 × 10^5^ cells/well and cultured in 2 ml/well of NGM or HGM. Sixteen hours after seeding, the cultures were treated with LPS (from *Escherichia coli* O55:B5, Cat#: L2880, Sigma‐Aldrich, stock concentration: 1 mg/ml, dissolved in saline) to the final concentration of 500 ng/ml or an equal volume of saline (vehicle control). To characterize the effects of HGM and LPS on molecules involved in LPS signaling pathways, we conducted a 2 (HGM or NGM) × 2 (with or without LPS) experiment design. The BV2 cells were collected before, and 0.5, 1, 2, 4, 8, 12, and 24 h after the LPS treatment. Each analysis contains three biological replicates obtained from three independent experiments.

### 2.2 Western blots

The BV2 cells were lysed in chilled RIPA buffer (Cat#: 89900, Thermo Fisher Scientific) containing protease inhibitors (Cat#: 04693116001, Roche) and phosphatase inhibitors (Cat#: 04906837001, Roche). The homogenates were centrifuged at 15,000×*g* for 15 min at 4°C. Protein concentrations of the supernatants were measured using a BCA Kit (Cat#: 23225, Thermo Fisher Scientific) and adjusted to the same. The supernatants (20 μg of total protein) were mixed with a sample buffer (Cat#: S3401, Sigma‐Aldrich) supplemented with 2% β‐mercaptoethanol (Cat#: 19‐1335, Sigma‐Aldrich), denatured by boiling, and resolved in polyacrylamide gels (8%–15%) at 110 V for 2 h. Twelve samples (4 groups × 3 biological replicates) collected at the same time point were loaded into the same gel. The separated proteins were transferred to PVDF membranes (Cat#: IPVH00010, Merck‐Millipore), blocked with 5% skim milk, and probed with respective primary antibodies: TLR4 (1:1000, Cat#: sc‐293072, Santa Cruz Biotechnology), Phospho‐p65 (1:1000, Cat#: 3033, Cell Signaling Technology), p65 (1:1000, Cat#: 8242, Cell Signaling Technology), iNOS (1:1000, Cat#: 13120, Cell Signaling Technology), Phospho‐p38 (1:1000, Cat#: 4511, Cell Signaling Technology), p38 (1:5000, Cat#: 8690, Cell Signaling Technology), Phospho‐Erk1/2 (1:1,000, Cat#: 4370, Cell Signaling Technology), Erk1/2 (1:5000, Cat#: 4695, Cell Signaling Technology), Phospho‐JNK (1:1000, Cat#: 4668, Cell Signaling Technology), JNK (1:1000, Cat#: 4668, Cell Signaling Technology), and α‐tubulin (1:10,000, Cat#: T9026, Sigma‐Aldrich) for 16 h at 4°C. After washing, the membranes were subsequently hybridized with proper horseradish peroxidase (HRP)‐conjugated secondary antibodies (goat anti‐mouse IgG: Cat#: 115‐035‐166; goat anti‐rabbit IgG: Cat#: 111‐035‐144, Jackson ImmunoResearch). The bound antibodies were detected using an enhanced chemiluminescence detection kit (Cat#: WBKLS0500, Merck‐Millipore) and X‐ray film (Cat# Super RX, Fujifilm). For re‐probing, the membranes were incubated with a stripping buffer containing 2% SDS, 62.5 mM Tris, and 0.8% β‐mercaptoethanol for 20 min at 55°C to remove the bound antibodies. The band densities were analyzed using ImageJ software (v2.0.0‐rc‐69/1.52p, U.S. National Institutes of Health). Relative protein expression was estimated by normalizing with the levels of the respective total form of protein or α‐tubulin. Band densities from one identical sample applied to each gel were used to normalize band densities among gels. The dilution ratios of antibodies and the image exposure time were tested to confirm that the luminescence signals were within the linear range of detection.

### 2.3 Statistical analysis

All numerical data are expressed as mean ± standard deviation. Statistical analyses and graph plotting were performed using the Prism software (v. 7.0a, GraphPad Software Inc.). Significance was set at *p* < .05. The differences of basal expression of selected proteins between NGM and HGM groups were analyzed with unpaired two‐tailed Student's *t*‐test. The relative expression of proteins of interest in each time point after the LPS treatment was analyzed by two‐way ANOVA followed by Tukey's multiple comparison tests if the main effects or interactions were significant. One‐way ANOVA followed by Dunnett's multiple comparisons was used to analyze the time‐dependent differences of relative expression of target proteins in the same group.

## 3 RESULTS

### 3.1 Effects of HGM and LPS on activations of NF‐κB and iNOS in BV2 microglial cells

**Figure 1:**
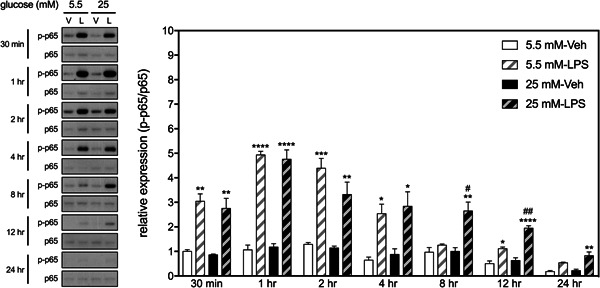
Effects of HGM and LPS on phosphorylation of p65 in BV2 microglial cell. Representative micrographs and corresponding quantitative results for Western blots. LPS, 500 ng/ml. Data are expressed as mean ± standard deviation. HGM, high glucose medium; LPS, lipopolysaccharide. *p < .05, **p < .01, ***p < .001, ****p < .0001, versus respective Veh group at the same time point, Tukey's multiple comparison test. #*p* < .05, ##*p* < .01, versus respective 5.5 mM group at the same time point, Tukey's multiple comparison test. *n* = 3

We first examined the effects of HGM on the LPS‐induced phosphorylation of p65 component, which is involved in the NF‐κB canonical pathway.^18^ Two‐way ANOVAs revealed that LPS, but not HGM, affected phosphorylation levels of p65 in BV2 cells at all selected time points within 24 h (Figure 1). Significant interactions between HGM and LPS were evident at 8‐ and 12‐h time points. Post hoc analyses revealed that LPS‐induced phosphorylation levels of p65 were higher in the HGM group than those of the NGM group at both time points (Figure 1).

**Figure 2:**
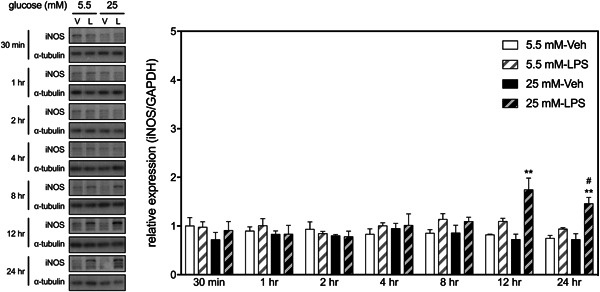
Effects of HGM and LPS on the expression of iNOS in BV2 microglial cells. Representative micrographs and corresponding quantitative results for Western blots. LPS, 500 ng/ml. Data are expressed as mean ± standard deviation. HGM, high glucose medium; iNOS, inducible nitric oxide synthase; LPS, lipopolysaccharide. **p < .01, versus respective Veh group at the same time point, Tukey's multiple comparison test. #*p* < .05, versus respective 5.5 mM group at the same time point, Tukey's multiple comparison test. *n* = 3

We then examined the interactive effects of HGM and LPS on the expression of iNOS, an inflammatory mediator whose expression in mammalian cells is predominantly governed by the NF‐κB.^19^ Two‐way ANOVAs revealed significant interactions between HGM and LPS on the levels of iNOS at 12‐ and 24‐h time points (Figure 2). Post hoc analyses revealed that only when BV cells cultured in HGM did LPS induce significant increases in iNOS expressions at these two time points. These results suggest that HGM prolongs LPS‐induced activation of p65, which subsequently upregulates the expression of iNOS in the BV2 microglial cells.

### 3.2 Effects of HGM and LPS on activations of MAP kinases in BV2 microglial cells

**Figure 3:**
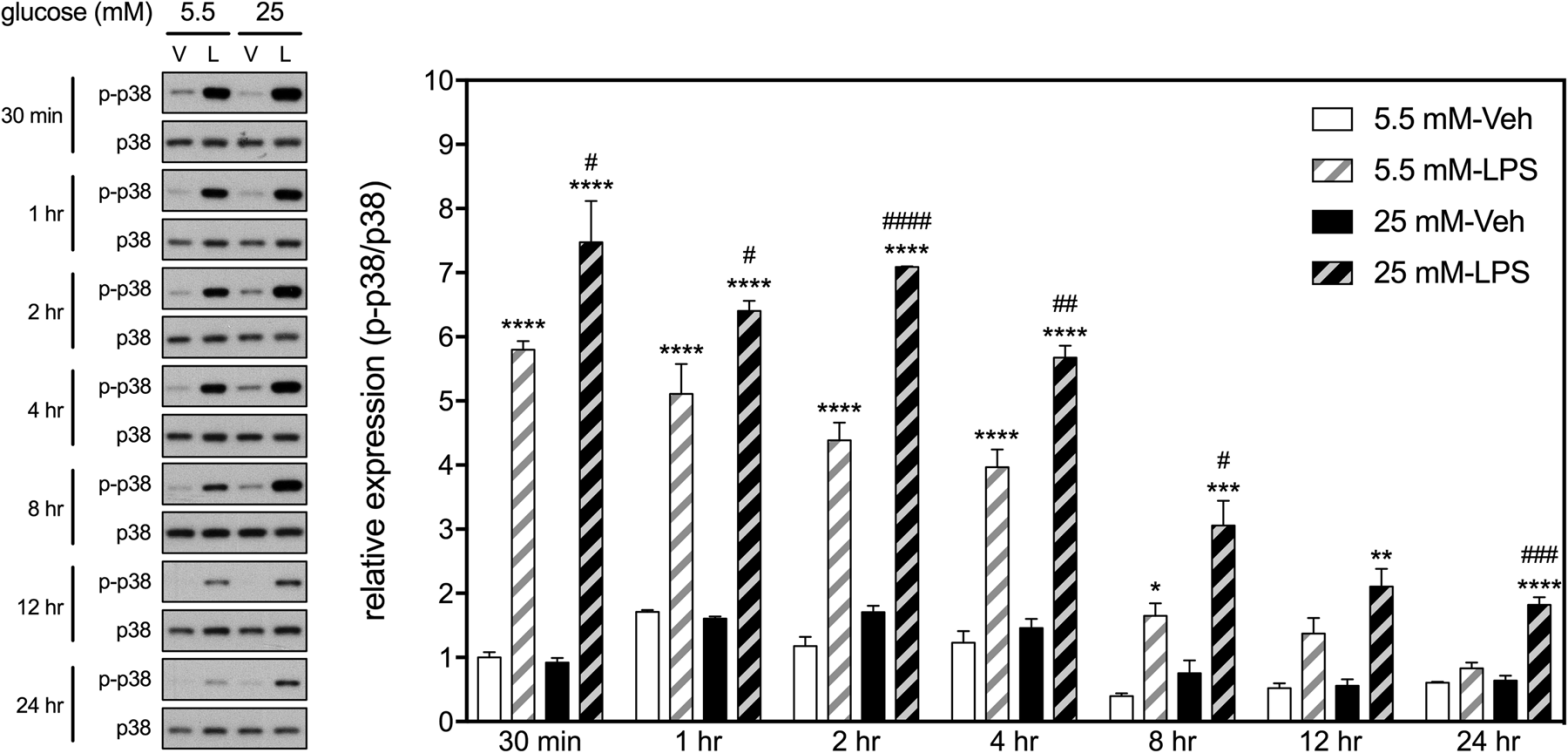
Effects of HGM and LPS on phosphorylation of p38 in BV2 microglial cells. Representative micrographs and corresponding quantitative results for Western blots. LPS, 500 ng/ml. Data are expressed as mean ± standard deviation. HGM, high glucose medium; LPS, lipopolysaccharide. *p < .05, **p < .01, ***p < .001, ****p < .0001, versus respective Veh group at the same time point, Tukey's multiple comparison test. #*p* < .05, ##*p* < .01, ###*p* < .001, ####*p* < .0001, versus respective 5.5 mM group at the same time point, Tukey's multi*p*le comparison test. *n* = 3

The effects of HGM on the LPS‐induced activations of three MAP kinases, p38, Erk1/2, and JNK in BV2 microglia were studied. Results showed that LPS induced phosphorylation of p38 within 30 min, which gradually tapered off as time increased (Figure 3). Post hoc analyses revealed that levels of LPS‐induced p‐p38 were significantly increased up to 8 h when cells grew in NGM. However, such effect remained significant up to 24 h when cells were cultured in HGM. LPS‐induced phosphorylation levels of p38 in the HGM group were higher than those of the NGM group at all time points, except 12 h.

**Figure 4:**
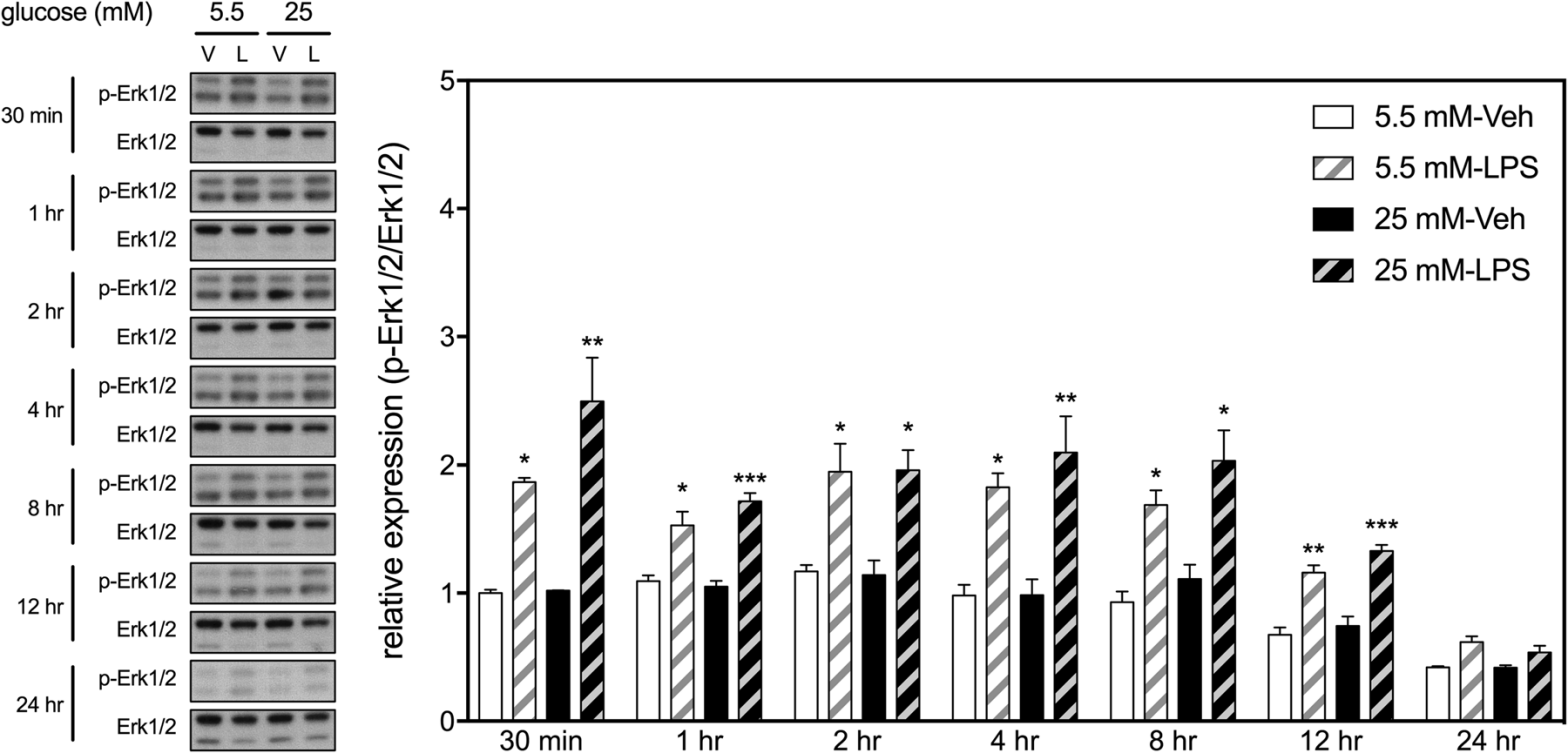
Effects of HGM and LPS on phosphorylation of Erk1/2 in BV2 microglial cells. Representative micrographs and corresponding quantitative results for Western blots. LPS, 500 ng/ml. Data are expressed as mean ± standard deviation. HGM, high glucose medium; LPS, lipopolysaccharide. *p < .05, **p < .01, ***p < .001, versus respective Veh group at the same time point, Tukey's multiple comparison test. *n* = 3

**Figure 5:**
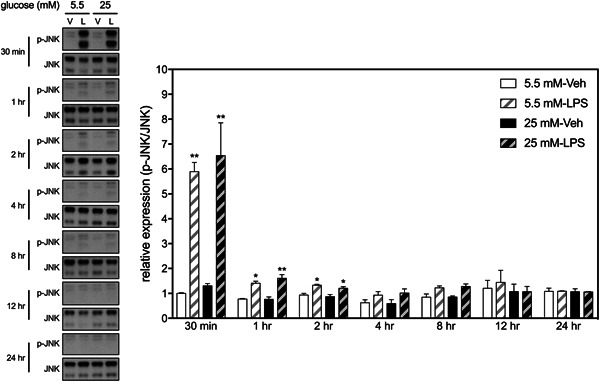
Effects of HGM and LPS on phosphorylation of JNK in BV2 microglial cells. Representative micrographs and corresponding quantitative results for Western blots. LPS, 500 ng/ml. Data are expressed as mean ± standard deviation. HGM, high glucose medium; LPS, lipopolysaccharide. *p < .05, **p < .01, versus respective Veh group at the same time point, Tukey's multiple comparison test. *n* = 3

LPS also induced phosphorylation of Erk1/2 within 30 min in BV2 cells, which resumed to basal levels 24 h later (Figure 4). There was no significant HGM effect or interaction between HGM and LPS on the expression of Erk1/2 at any selected time points. Likewise, LPS induced phosphorylation of JNK within 30 min in BV2 cells but rapidly resumed to basal levels within 4 h (Figure 5). There was no significant HGM effect or interaction between HGM and LPS on the expression of JNK at any selected time points. These results suggest that HGM differentially regulated the LPS‐induced activation of p38 signaling pathways without affecting the other two MAP kinase (i.e., Erk1/2 and JNK) pathways.

### 3.3 Effects of HGM and LPS on expressions of TLR4 in BV2 microglial cells

**Figure 6:**
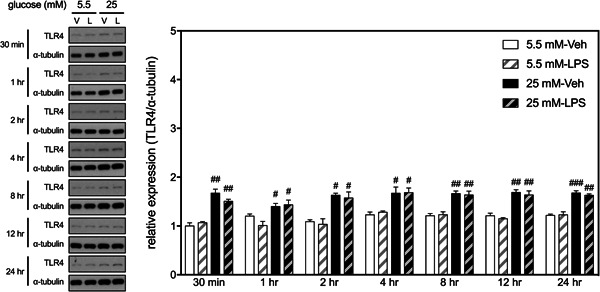
Effects of HGM and LPS on expressions of TLR4 in BV2 microglial cells. Representative micrographs and corresponding quantitative results for Western blots. LPS, 500 ng/ml. Data are expressed as mean ± standard deviation. HGM, high glucose medium; LPS, lipopolysaccharide. #*p* < .05, ##*p* < .01, ###*p* < .001, versus respective 5.5 mM group at the same time point, Tukey's multiple comparison test. *n* = 3

To search for a potential factor that may cause the inflammatory enhancing effect of HGM, we determined the interactive effects of HGM and LPS on the expression of TLR4, the major LPS receptor expressed by cells of the innate immune system.^16^ Two‐way ANOVAs revealed that, at all selected time points, HGM, but not LPS, affected levels of TLR4 in BV2 cells (Figure 6). There was no significant interaction between HGM and LPS at any time point. Post hoc analyses also indicated that levels of TLR4 in two HGM groups were significantly higher than their respective control (NGM) groups at all selected time points.

## 4 DISCUSSION

Upon inflamagen stimulation, microglia undergo activation and release several pro‐inflammatory mediators, including cytokines, nitric oxide, and reactive oxygen species. These responses are required to protect the host against infections and injuries. However, under certain pathological conditions, the acute inflammatory response may progress to chronic inflammation. Accumulations of these pro‐inflammatory mediators are harmful to the neighboring neurons and cause further microglial activation, leading to a vicious cycle.^20^ Hence, controlling microglia‐mediated neuroinflammation is critical to prevent further neuronal injuries. Here, we showed that HGM prolonged LPS‐induced activation of NF‐κB, subsequently leading to increased expressions of iNOS in BV2 microglial cells. Furthermore, HGM aggravated the LPS‐induced activation of p38 signaling without affecting the activation of the other two MAP kinases, Erk1/2 and JNK. These potentiation effects may be derived from HGM‐related increases in TLR4 expression levels.

High‐glucose culturing conditions increase expressions of TLR4 level have been reported previously in cell types other than BV2 microglial cells. Comparing human THP‐1 monocytes cultured in media with three different glucose concentrations (i.e., 5.5, 15, and 25 mM), Dasu et al.^21^ showed that levels of TLR2 and TLR4 were increased in a dose‐dependent manner. High glucose‐induced upregulation of TLR4 has also been shown in endothelial cells.^22^ By using pharmacological and genetic approaches, two independent groups have demonstrated that PKC, especially PKC‐δ, is involved in mediating the high glucose‐induced upregulation of TLR4.^21^, ^23^ Furthermore, increased NF‐κB activities were observed in THP‐1 monocytes cultured in high‐glucose media.^21^ The high glucose‐induced activation of NF‐κB and the productions of pro‐inflammatory cytokines and chemokines in THP‐1 monocytes could be ameliorated by knocking down of TLR4.^21^ However, in this study, we failed to notice an increased activation of NF‐κB in BV2 cells cultured in HGM alone. These results suggest that microglia may have a higher tolerance to glucose than monocytes/macrophages. Interestingly, we found that high glucose prolonged LPS‐induced activation of NF‐κB, rather than amplifying it. That is, the enhancing effect of HGM was unnoticed until 8 h later. The prolonging effect may be derived from reduced internalization of TLR4 from the cell plasma membrane and/or shifted the balance between kinase/phosphatase activities that regulate the IKKβ‐NF‐κB activation.^24^

In this study, we showed that under LPS stimulation, high glucose enhanced the activations of p38 without affecting the activation statuses of Erk1/2 or JNK in the BV2 microglial cells. The activation of p38 pathway is known to regulate LPS‐induced productions of pro‐inflammatory cytokines, such as tumor necrosis factor, interleukin‐1β, interleukin‐6, interleukin‐8, and interleukin‐10.^25^, ^26^, ^27^, ^28^ Overexpression of these pro‐inflammatory cytokines has been implicated in developments of neuroinflammation‐related neuronal excitotoxicity,^29^ apoptosis,^30^ and neurodegeneration diseases,^31^ which are known to be affected by diabetes mellitus.^32^, ^33^, ^34^, ^35^, ^36^ Therefore, the TLR4/p38 pathway may be a potential therapeutic target for controlling hyperglycemia‐associated microglia activation and neuroinflammation.

It is worth mentioning that we did not observe a high‐glucose effect on the basal expressions of MAP kinases in the BV2 microglial cells. However, several previous studies showed that high glucose alone was capable of activating the p38 pathway in smooth muscle cells,^37^ endothelium,^38^ and mesothelium.^39^ Whether this discrepancy is caused by different culture conditions (e.g., glucose concentrations, treatment duration, etc.) and/or a cell type‐specific phenomenon deserves further investigation.

## 5 CONCLUSION

Environmental glucose levels affect the LPS signaling pathways in microglia. High glucose enhanced the LPS‐induced activation of the NF‐κB/iNOS pathway by prolonging p65 phosphorylation and elevating iNOS expression in BV2 cells. High glucose also aggravated the LPS‐induced activation of p38 signaling without affecting the Erk1/2 and JNK signaling pathways. These prolonging and aggravating effects may be derived from high glucose‐mediated upregulation of TLR4. Controlling blood glucose levels will be a beneficial means to manage neuroinflammation and related neurodegenerative diseases.

## CONFLICTS OF INTEREST

The authors declare no conflicts of interest.

## Data Availability

All data sets generated or analyzed in this study were included in the published article. Detailed data sets supporting the current study are available from the corresponding authors upon request
